# Overview of extracellular vesicles as biomarkers and therapeutic tools in pediatric diseases: focus on the gut-lung axis

**DOI:** 10.20517/evcna.2025.88

**Published:** 2025-12-04

**Authors:** Paola Bisaccia, Alice Zaramella, Agner Henrique Dorigo Hochuli, Raquel Moll Diaz, Miriam Duci, Maurizio Muraca, Eugenio Baraldi, Michela Pozzobon

**Affiliations:** ^1^Department of Women’s and Children’s Health, University of Padova, Padova 35129, Italy.; ^2^Foundation, Institute of Pediatric Research Città della Speranza, Padova 35127, Italy.

**Keywords:** Gut, lung, extracellular vesicles, inflammatory bowel disease, necrotizing enterocolitis, bronchopulmonary dysplasia, chronic obstructive pulmonary disease

## Abstract

Although anatomically separate, the gut and lungs are interconnected through intricate pathways involving their respective microbiota, supporting the concept of a gut-lung axis. In the pediatric field, devastating intestinal pathologies such as necrotizing enterocolitis and inflammatory bowel diseases mostly affect preterm infants. In parallel, in the lung, bronchopulmonary dysplasia and chronic obstructive pulmonary disease represent pediatric unmet clinical needs. In this review, we discuss how the extracellular vesicles (EVs), nanoparticles secreted by all cell types, represent a common element in the gut-lung axis. Specifically, EVs play a dual role, serving both as novel disease biomarkers and as promising therapeutic agents.

## INTRODUCTION

In recent years, the concept of intercellular communication has undergone a profound transformation with the discovery and characterization of extracellular vesicles (EVs), which are small, membrane-bound particles actively secreted by nearly all cell types. While in the past they were considered cellular garbage, EVs are now recognized as functional cell-cell messengers that carry a wide array of bioactive molecules, including proteins, lipids, messenger RNAs (mRNAs), microRNAs (miRNAs) and even DNA fragments^[1,2]^. These vesicles modulate the behavior of recipient cells, becoming key players in local and systemic biological processes^[3]^. EVs are broadly categorized based on their size, surface markers and biogenesis into three main classes: exosomes, microvesicles, and apoptotic bodies^[4]^.

Each class arises from distinct intracellular pathways and carries specific cargo reflective of its cellular origin and the physiological or pathological state of the parent cell. The selective packaging of this cargo is not random, but rather a tightly regulated process that enables targeted communication and functional specificity^[5]^. The ability of EVs to transfer complex information from one cell to another has established them as crucial mediators in a broad spectrum of physiological functions, including immune regulation, tissue development, cellular homeostasis, and inter-organ communication^[5,6]^. EVs in fluids, such as in peripheral blood, urine and saliva, are valuable as disease biomarkers and prognostic indicators of treatment response or disease progression. EVs hold significant potential in pediatric diseases, given that many childhood conditions involve intricate developmental processes and can have lasting impacts on growth and organ function. EVs, as a therapeutic agent, may provide a less invasive and potentially more effective alternative to existing treatment approaches.

Beyond their physiological functions, EVs are also involved in various pathological conditions, playing a paramount role in disease progression. Concerning inflammatory diseases, EVs can either propagate inflammation or act as regulatory agents that limit tissue damage^[7,8]^. The dual nature of EVs, which can serve as both disease vectors and potential therapeutic tools, has generated considerable interest in their application as biomarkers, therapeutic targets, and drug delivery systems^[9,10]^.

One particularly intriguing and emerging area of EV research is their role in mediating communication between distant organs. A notable example of this systemic interaction is the gut-lung axis, a bidirectional communication network between the gastrointestinal and respiratory systems. Although these organs are anatomically distinct and functionally specialized, research suggests that they share immune, neural, and microbial connections that allow them to influence each other’s physiological state^[11,12]^. EVs represent the natural connection among organs. Specifically, this review will focus on the communication between the gastrointestinal and respiratory systems. The crosstalk between the gut and the lungs, indeed, is mediated not only by circulating immune cells and microbial metabolites but also by EVs^[13]^.

These nanoparticles carry signals capable of modulating inflammation, barrier integrity, and immune responses in patients with gastrointestinal inflammation and respiratory complications^[14,15]^.

**Figure 1:**
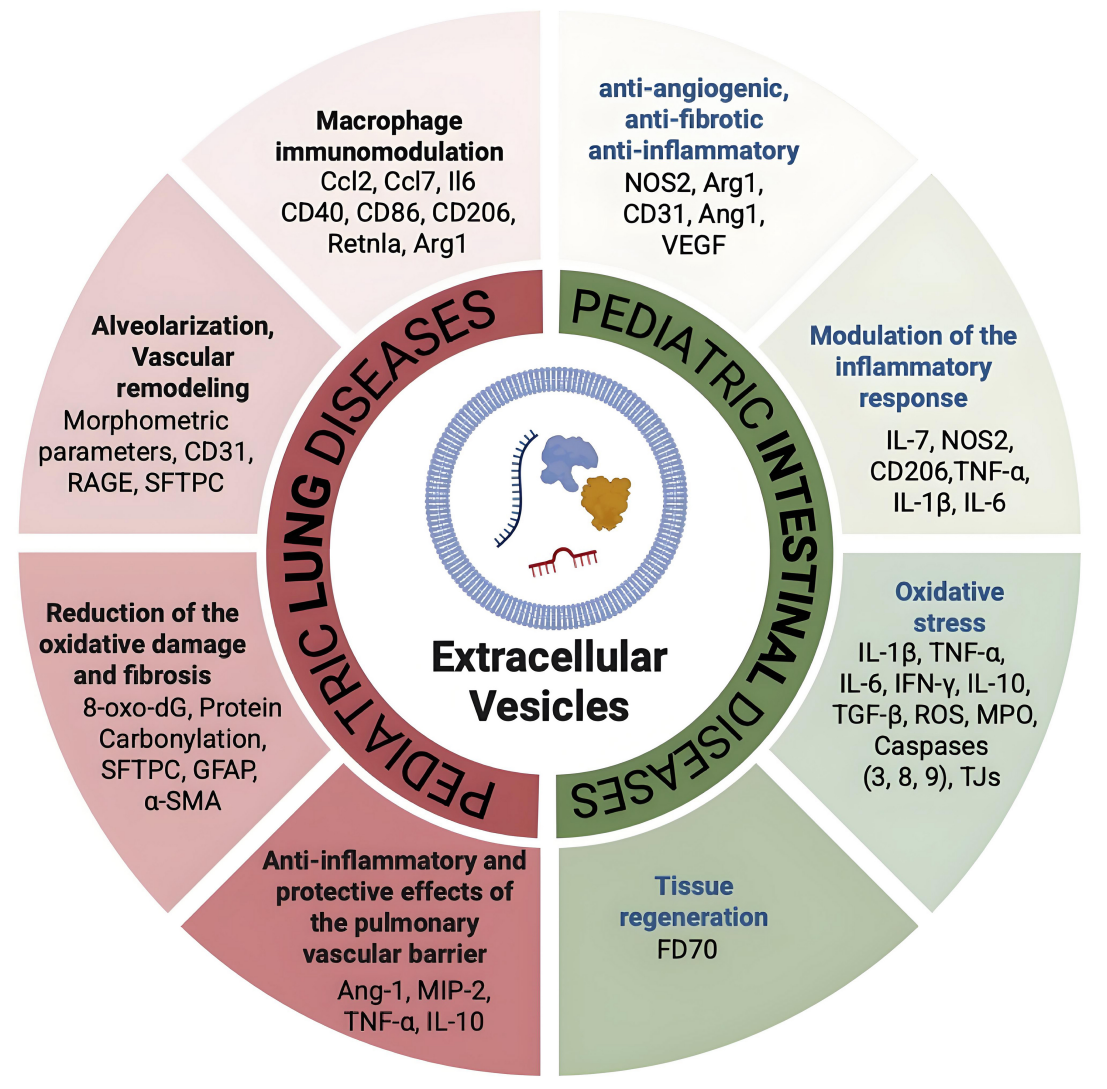
Summary of the EV molecular targets in diseased intestine and lung. The multifaceted target molecules of EVs are depicted in this cartoon: EVs, as one important natural bridge between intestine and lung, act by modulating compartment-specific molecules but also common molecules such as TNF-α and macrophage markers (CD80, CD86, Arg1, NOS2). [Created with BioRender. Dorigo Hochuli, A. (2025) https://BioRender.com/awjluhs]. IL-1β: Interleukin-1 beta; IL-6: interleukin-6; IL-7: interleukin-7; IL-10: interleukin-10; IFN-γ: interferon gamma; TNF-α: tumor necrosis factor alpha; TGF-β: transforming growth factor beta; NOS2: nitric oxide synthase 2; Arg1: arginase 1; Ang-1: angiopoietin-1; CD31: cluster of differentiation 31; CD40: cluster of differentiation 40; CD86: cluster of differentiation 86; CD206: mannose receptor CD206; CCL2: C-C motif chemokine ligand 2; CCL7: C-C motif chemokine ligand 7; MIP-2: macrophage inflammatory protein-2; RAGE: receptor for advanced glycation end products; SFTPC: surfactant protein C; GFAP: glial fibrillary acidic protein; α-SMA: alpha-smooth muscle actin; ROS: reactive oxygen species; MPO: myeloperoxidase; TJs: tight junctions; 8-oxo-dG: 8-oxo-2’-deoxyguanosine; EVs: extracellular vesicles; FD70: ﬂuorescein isothiocyanate-dextran 70; RetnIa: resistin-like alpha.

In summary, EVs are increasingly recognized as promising tools for both therapeutic and diagnostic applications, marking a pivotal advancement in biomedical research. Since their relatively recent identification as regulators of both physiological and pathological processes, there has been a rapid expansion of interest in their clinical utility. Their intrinsic ability to transfer bioactive molecules between cells highlights their potential to influence disease progression and tissue homeostasis [Table 1 and Figure 1]. As a result, EV-based strategies for diagnosis and therapy are rapidly evolving, positioning these vesicles at the forefront of next-generation medical innovations. Accordingly, ongoing and completed clinical trials applying EVs as a therapeutic tool in pediatric diseases are summarised in Table 2. In this manuscript, we present the currently known aspects of understanding the role of EVs within the gut-lung axis. Studies on the systemic nature of mucosal immunity and the pathogenesis of chronic inflammatory diseases will open new avenues for therapeutic interventions aimed at harnessing or modifying EV-mediated communication to restore homeostasis across organ systems. In this review, we examine current evidence for EV involvement in the communication between the lung and intestine (EVs as biomarkers), highlighting the implications for disease pathophysiology and future clinical applications (EVs as a therapeutic tool). As summarized in the graphical abstract, regarding the gut, we will focus on necrotizing enterocolitis (NEC) and inflammatory bowel diseases (IBD); at the same time, for the lung, we will consider bronchopulmonary dysplasia (BPD) and chronic obstructive pulmonary disease (COPD).

**Table 1:** Summary of preclinical studies evaluating the therapeutic effects of EVs derived from various stem cell sources in models of inflammatory and degenerative pediatric diseases

**Model**	**Cell source of EVs**	**Dose of EVs**	**Number of administration**	**Route of administration**	**Therapeutic mechanism**	**Molecular target as a biomarker**	**Reference**
IBD	MSC from umbilical cord	1.3 × 10^6^	3	IV (tail vein)	Modulation of the inflammatory response	IL-7, NOS2, CD206, TNF-α, IL-1β, IL-6	[29]
	IFN-γ-primed bone marrow- MSC	200 µg	Single dose	IV	Inhibition of Stat3 via miR-125a/b	N.A.	[30]
	MSC (murine source)	nEV: 1.10^9^ iEV: 1.10^9^	5	IP	iEV: immunomodulation, anti-angiogenic, anti-fibrotic effects Tissue regeneration	NOS2, Arg1, CD31, Ang1, VEGF	[31]
	Human placental MSC	200 µg	Single dose	*In situ* administration in the site of injury (colon mesangial margin)	Inhibition of inflammation and oxidative stress	IL-1β, TNF-α, IL-6, IFN-γ, IL-10, TGF-β, ROS, MPO, Caspases (3, 8, 9), Tight junction proteins (Claudin-1, ZO-1, Occludin)	[32]
NEC	Bovine milk-derived exosomes	1 µg/µL	3 doses/day (from P5 to P9)	Gavage	Modulation of NLRP3 inflammasome and NF-κB signaling in the lung during neonatal necrotizing enterocolitis	MPO, IL-1β, P-NF-κB/NF-κB ratio	[34]
	Bone marrow- MSC	2.5 × 10^9^	Single dose at 5 h after delivery	IP	Preservation of gut barrier function wound healing of IEC-6 cells promotion *in vitro*	FD70	[41]
BPD	Wharton’s Jelly and bone marrow MSC	Bolus dose	Single dose at P4	IV	Macrophage immunomodulation	Ccl2, Ccl7, IL-6 CD40, CD86 CD206, Retnla, Arg1	[72]
	Wharton’s Jelly MSC	EV dose/g body wt: 8 × 10^8^ at P3, 4.5 × 10^8^ at P7, and 3 × 10^8^ at P10	3 doses: P3, P7, P10	IT	Promotion of the alveolarization and of the vascular remodelling Inhibition of the fibrotic process Immunomodulation	Morphometric parameters	[73]
	Wharton’s Jelly MSC	6,4 × 10^9^	3 doses: P3, P7, P10	IT	Reduction of oxidative damage and fibrosis	8-oxo-dG, protein carbonylation, SP-C, GFAP, α-SMA	[74]
	Human bone marrow MSC	2 × 10^11^	2 doses: 6 and 72 h after delivery	IV	Promotion of alveolarization and vascularization, reduction of airway and vascular smooth muscle thickening, improvement of gas exchange VEGF-R2	SP-B, PCNA, caspase-3	[75]
	Wharton’s Jelly MSC	2 × 10^8^ EV/g body wt	Single dose at P3	IT, IV	Reduction of inflammation, angiogenesis restoration, prevention of long-term cardiopulmonary damage	VEGF, CXCL12, CXCR4, IL-1β, IL-6, TNF-α	[76]
	Bone marrow MSC	800 μg	Single dose	IV (tail vein)	Therapeutic tool miR-425 Delivered via exosomes to suppress PTEN and activate the PI3K/AKT pathway Therapeutic target: PTEN Target of miR-425, inhibited to reduce apoptosis	miR-425 Its levels indicate disease status and treatment effectiveness	[77]
COPD	Human bone marrow-derived MSCs - MVs	10 µL of MVs released by 1 × 10^6^ cells	Single dose	IT	Anti-inflammatory and protective effects on the pulmonary vascular barrier Ang-1	Ang-1, MIP-2, TNF-α, IL-10	[78]
	Human placenta MSC	0.1 mL	5 days per week for 3 weeks	IP	Restoration of lung function and inflammation reduction. Suppression of pro-inflammatory immune cells, promotion of anti-inflammatory response	N.A.	[80]

EVs: Extracellular vesicles; MSC: mesenchymal stem cell; IFN-γ: interferon gamma; nEV: naïve extracellular vesicles; iEV: induced extracellular vesicles; IL-7: interleukin-7; NOS2: nitric oxide synthase 2; CD206: cluster of differentiation 206; TNF-α: tumor necrosis factor-alpha; IL-1β: interleukin-1 beta; IL-6: interleukin-6; miR: microRNA; Arg1: arginase 1; CD31: cluster of differentiation 31; Ang1: angiopoietin 1; VEGF: vascular endothelial growth factor; IL-10: interleukin-10; TGF-β: transforming growth factor beta; ROS: reactive oxygen species; MPO: myeloperoxidase; ZO-1: zonula occludens-1; NEC: necrotizing enterocolitis; NF-κB: nuclear factor-kappa B; P-NF-κB: phosphorylated nuclear factor-kappa B; IEC: intestinal epithelial cell; FD70: fluorescein isothiocyanate-dextran 70; BPD: bronchopulmonary dysplasia; Ccl: C-C motif chemokine ligand; Retnla: resistin-like alpha; SP: surfactant protein; GFAP: glial fibrillary acidic protein; α-SMA: alpha-smooth muscle actin; PCNA: proliferating cell nuclear antigen; CXCL12: C-X-C motif chemokine ligand 12; CXCR4: C-X-C chemokine receptor 4; PTEN: phosphatase and tensin homolog; PI3K/AKT: phosphoinositide 3-kinase/protein kinase B; COPD: chronic obstructive pulmonary disease; MVs: microvesicles; MIP-2: macrophage inflammatory protein 2; IV: intravenous; IP: intraperitoneal; IT: intratracheal; STAT3: signal transducer and activator of transcription 3; NLRP3: NLR family pyrin domain containing 3; VEGF-R2: vascular endothelial growth factor receptor 2; CD40: cluster of differentiation 40; CD86: cluster of differentiation 86; 8-oxo-dG: 8-Oxo-2’-deoxyguanosine; N.A.: Not Applicable.

**Table 2:** Overview of selected clinical trials using EVs derived from stem cells for the treatment of pediatric diseases

**Disease**	**Source of EVs**	**Name of the preparation**	**Clinical trial ID**	**Route of administration**	**Status of the trial**
BPD	Human bone marrow MSC	UNEX-42	NCT03857841	Intravenous	Terminated
	Human Umbilical Cord MSC	EXOB-001	NCT06279741	Endotracheal	Recruiting
IBD	Human bone marrow MSC	ExoFlo	NCT05130983	Intravenous	Terminated

The table shows the target disease, the source and name of the EV preparation, clinical trial ID, route of administration, and current trial status. The trials primarily focus on BPD and IBD. Data were obtained from the NIH Clinical Trials Database (https://clinicaltrials.gov/). EVs: Extracellular vesicles; MSC: mesenchymal stem cell; BPD: bronchopulmonary dysplasia; IBD: inflammatory bowel disease; NIH: National Institutes of Health; ID: identifier.

Table 1 provides an overview of experimental studies exploring the therapeutic potential of EVs isolated from different mesenchymal stromal/stem cell (MSC) sources—including human umbilical cord, placenta, bone marrow (BM), Wharton’s Jelly (WJ), and bovine milk—in multiple preclinical models of pediatric diseases such as IBD, necrotizing enterocolitis (NEC), BPD, COPD. Reported parameters include the disease model, EV source, dosage, number and timing of administrations, and route of injection [intravenous (IV), intraperitoneal (IP), intratracheal (IT), oral gavage, or local *in situ* delivery at the site of injury]. The column “Therapeutic mechanism” outlines the key mechanisms by which EVs exert their therapeutic effects, including immune modulation, inhibition of inflammation, oxidative stress and fibrosis, and promotion of tissue repair and barrier integrity. The “Molecular Target as a Biomarker” summarizes molecular indicators used to evaluate efficacy, such as markers of inflammation, oxidative stress, apoptosis, epithelial integrity, and angiogenesis. Overall, EVs demonstrated multiple benefits across models, supporting their translational potential as a cell-free therapeutic strategy for pediatric inflammatory and degenerative diseases.

## GUT-LUNG AXIS: THE ROLE OF EVs

### Pediatric gastrointestinal diseases

The disruption of intestinal homeostasis caused by impairment of the intestinal barrier is common to two pediatric gastrointestinal disorders: NEC and IBD^[16]^. The gut-lung axis describes the bidirectional communication between the gastrointestinal and respiratory systems, mediated by the immune system, microbial metabolites, and soluble mediators^[17,18]^. Dysregulation of this axis contributes to extra-intestinal manifestations in IBD and systemic complications in NEC, such as lung injury and impaired alveolar development^[19]^. NEC is the most prevalent severe gastrointestinal condition seen in neonatal intensive care units. It is a significant cause of morbidity and mortality among newborns, especially in premature infants^[20]^.

Studies in neonatal mice have shown that at the cellular level, a key event preceding widespread systemic inflammation is the activation of Toll-like receptor 4 (TLR4) on the intestinal epithelium by bacterial lipopolysaccharides (LPS). TLR4 activation triggers enterocyte apoptosis and the release of High Mobility Group Box 1 (HMGB1), which amplifies inflammation by perpetuating TLR4 signaling in distant organs. As HMGB1 enters systemic circulation, it reaches the lungs, where TLR4 activation on pulmonary epithelial cells induces a pro-inflammatory response. This leads to nuclear translocation of the nuclear factor kappa-light-chain-enhancer of activated B cells (NF-κB) and the upregulation of cytokines such as pro-interleukin (IL)-1β (pro-IL-1β), priming the Nucleotide-binding oligomerization domain-containing protein 2 (NOD)-like receptor family pyrin domain-containing 3 (NLRP3) inflammasome. The resulting cytokine storm contributes to lung injury, a process implicated in the development of chronic lung disease in NEC survivors^[16,17]^. Interestingly, the open field of engineered EVs demonstrated that EVs modified with Heme Oxygenase-1 (HO-1) can convey microRNA-200b to intestinal epithelial cells, thereby suppressing HMGB3-mediated inflammatory responses^[18]^.

IBD represents a global healthcare challenge with rising incidence, characterized by chronically relapsing intestinal inflammation. The two main types are Crohn’s disease (CD) and ulcerative colitis (UC), which differ in the extent and localization of inflammation^[19]^. This disruption not only affects intestinal homeostasis but also has systemic effects on the patient’s overall health. Patients with IBD often exhibit pulmonary manifestations, including bronchitis, interstitial lung disease, and airway inflammation. The shared embryonic origin of the gut and lung, alongside systemic immune priming and microbial translocation, contributes to these effects. Animal models of colitis demonstrate altered pulmonary cytokine profiles^[20]^, underlining the crosstalk between the gut and lung (gut-lung axis). Briefly, gut dysbiosis - an imbalance in the composition or function of the gut microbiota - can significantly alter gut anatomy, physiology, and immune responses. Intestinal inflammation resulting from this dysbiosis can compromise the epithelial barrier, allowing the translocation of gut contents, including microbes, immune cells, and pro-inflammatory molecules, into distant organs such as the lungs, where they can trigger or worsen inflammatory responses. Finally, immune cell mis-homing refers to the phenomenon where immune cells that are activated in the gut and commonly express gut-specific homing receptors, such as alpha 4 beta 7 (α4β7) integrin and C-C chemokine receptor type 9 (CCR9), migrate inappropriately to other tissues - most notably the lungs. This misdirection occurs due to disrupted chemokine signaling or altered expression of adhesion molecules, often in the context of systemic inflammation. When these gut-primed immune cells infiltrate the lungs, they may fail to perform their intended protective roles and instead promote inappropriate inflammation. This contributes to the breakdown of immune tolerance in the lungs and exacerbates chronic respiratory diseases, such as asthma and COPD, by altering the balance of local immune responses, which leads to tissue damage and disease progression^[21,22]^. Notably, regarding the intestine, the therapeutic efficacy of unmodified (naïve) EVs remains well supported, as MSC-derived EVs have been shown to significantly reduce disease activity, histopathological damage, oxidative stress, and levels of pro-inflammatory cytokines such as Tumor Necrosis Factor alpha (TNF-α), IL-6, and IL-1β, while concurrently enhancing the expression of the anti-inflammatory cytokine IL-10^[23]^.

### EVs as biomarkers in inflammatory bowel disease and NEC

IBD and NEC represent significant challenges in pediatric gastroenterology, primarily characterized by intestinal inflammation and epithelial injury. Different studies suggest that EVs may play a pivotal role not only in the pathogenesis but also as promising non-invasive biomarkers for diagnosis. In 2015, Leoni *et al*. observed the presence of annexin A1 (ANXA1) in EVs derived from intestinal epithelial cells and their ability to activate wound repair pathways^[24]^. ANXA1 is recognized for its anti-inflammatory properties in the gut, limiting leukocyte recruitment by inhibiting neutrophil migration and promoting apoptosis, acting downstream of glucocorticoids and mimicking many of their effects. Elevated serum levels of ANXA1-containing EVs were also observed when comparing healthy controls and patients with active inflammation due to IBD, indicating that ANXA1-containing EVs are systemically distributed in response to the inflammatory process. Therefore, a high level of ANXA1 could potentially serve as a biomarker of intestinal mucosal inflammation.

As previously mentioned, EVs were found in many fluids, including urine and saliva samples. The involvement of the oral cavity in IBD has already been documented^[25]^. Saliva from IBD patients contains characteristic EVs that may reflect the presence and development of IBD, potentially serving as biomarkers. Indeed, the isolation of EVs from the salivary samples of IBD patients and healthy controls revealed a distinct protein profile. Specifically, in 2017, Zheng *et al*. observed that eight proteins were uniquely present in patients with IBD. Among the eight, the proteasome subunit alpha type 7 (PSMA7) was found to be highly expressed in both patients with UC and CD^[26]^. This study also investigated the presence of this protein in an animal model. Animal experiments revealed that the expression level of PSMA7 in oral epithelial tissue was comparable to that in intestinal inflammation, suggesting its potential as a non-invasive diagnostic tool for identifying IBD patients.

Interestingly, a recent study detected EVs in the urine of premature neonates and revealed significantly altered miRNA profiles in those with NEC compared to healthy, age-matched controls. Notably, the differentially expressed miRNAs included miRNA-5703, miRNA-604, miRNA-5186, and miRNA-139-3p^[27]^. However, so far, no early biomarkers have been identified for these premature infants, and analyses are still ongoing^[28]^.

### EVs as therapeutic tool in inflammatory bowel disease and NEC

In preclinical models of IBD, particularly those induced by dextran sulfate sodium (DSS) and trinitrobenzene sulfonic acid (TNBS), mesenchymal stromal cell-derived EVs (MSC-EVs) have been shown to be potent immunomodulatory and regenerative agents. Their beneficial effects are multifaceted, involving the modulation of key inflammatory signaling pathways, the regulation of immune cells, and mucosal repair. Mao *et al*. demonstrated that human umbilical cord MSC-EVs reduced NF-κB p65 expression and nuclear translocation in DSS-induced colitis in mice, resulting in a marked decrease in IL-6 and TNF-α levels^[29]^. Yang *et al*. reported that BM MSC-EVs inhibited the phosphorylation of signal transducer and activator of transcription 3 (STAT3) in colonic epithelial cells and immune cells, leading to a reduced expression of downstream cytokines, such as IL-17A and IL-22^[30]^. Tolomeo *et al*. demonstrated the higher efficacy of naïve EVs (nEVs) and induced EVs (iEVs) from BM MSCs compared to MSCs alone in downregulating inflammation and modulating macrophage polarization toward alternatively activated (M2) macrophages^[31]^.

Under the anti-inflammatory properties, in the TNBS-induced colitis model, placenta tissue-derived MSC-EVs significantly reduced levels of IL-6 and TNF-α, contributing to the amelioration of disease severity and improved histological architecture^[32]^. These EVs also downregulated the expression of IL-17A, a key cytokine in T helper 17 cells (Th17)-mediated colitis, indicating a role in rebalancing adaptive immune responses^[33]^. Moreover, bovine milk-derived EVs can attenuate intestinal inflammation by modulating key inflammatory pathways (e.g., TLR4-NF-κB-NLRP3), promoting the polarization of macrophages towards an anti-inflammatory M2 phenotype, and stimulating epithelial repair mechanisms^[34]^.

miRNA cargo within EVs, such as miR-146a and miR-155, plays a pivotal role in immune modulation by targeting NF-κB signaling pathways and other key inflammatory mediators^[35]^. Additionally, proteomic analyses of EVs reveal enrichment of anti-inflammatory and tissue repair-associated proteins, including transforming growth factor beta (TGF-β) and IL-10, further supporting their immunosuppressive and regenerative capacities^[35]^. Although the therapeutic potential of EVs in preclinical models of NEC and IBD is promising, several challenges remain for their clinical use. These include standardizing EV isolation methods, scaling up production, and characterizing their bioactive contents. However, the growing evidence suggests that EVs provide a feasible and potentially effective alternative to cell-based therapies for IBD.

Multiple studies have demonstrated the efficacy of EVs in experimental NEC models. IP or IV administration of amniotic fluid-, BM-, or umbilical cord, breast milk-derived-EVs has been shown to reduce intestinal necrosis and histological injury, suppress pro-inflammatory cytokine production (e.g., IL-6, TNF-α, IL-1β), inhibit apoptosis and oxidative stress, restore epithelial integrity and tight junction (TJ) proteins (e.g., zonuline, occludin)^[36-39]^. Good *et al*. demonstrated that BM-derived EVs from amniotic fluid stem cells significantly decreased NEC severity and improved survival in a mouse model. The beneficial effects were linked to modulation of TLR4 signaling, a key inflammatory pathway implicated in NEC pathogenesis^[40]^. Similarly, studies by Rager *et al*. reported that EVs modulate tissue repair and the resolution of inflammation, playing a protective role in NEC onset^[41,42]^. As previously outlined, NEC is linked to systemic inflammation that can hinder lung development. Bovine-milk-derived EVs added to formula can decrease inflammasome activation, NF-κB pathway activity, and damage in the NEC lung, demonstrating their potential to restore the balance of the gut-lung axis^[39]^.

### Pediatric lung diseases

BPD, a complication of preterm birth, and COPD are both characterized by chronic pulmonary inflammation, epithelial dysfunction, and increased susceptibility to infection. Growing evidence suggests that the gut-lung axis, a system of immunological and microbial crosstalk between the gastrointestinal and respiratory tracts, plays a pivotal role in shaping pulmonary immunity in both diseases. Furthermore, EVs have emerged as key mediators of systemic communication, transporting cytokines, miRNAs and microbial components between the gut and lung compartments^[43]^. Understanding how the gut-lung axis and EVs intersect in BPD and COPD may unlock novel strategies for early intervention.

BPD is a common respiratory disorder affecting extremely preterm infants, associated with high morbidity and mortality. Newborns suffering from this chronic lung disease of multifactorial etiology present an undeveloped antioxidant system and very immature lungs, which lead to further complications that compromise their lives^[42]^. Since BPD may contribute to the development of COPD, it is crucial to reduce its burden to prevent long-term respiratory complications in patients suffering from the disease. There is evidence supporting various strategies to alleviate this burden. Notable approaches include surfactant therapy, vitamin A supplementation, protective non-invasive ventilation, and fluid restriction; however, these do not appear to have a significant impact on reducing the burden of BPD^[42,44]^. BPD can increase the risk of developing asthma, a chronic inflammatory airway disorder involving reversible airflow obstruction, airway hyperresponsiveness, and structural remodelling^[42]^.

COPD is a progressive respiratory condition marked by persistent airflow limitation, chronic inflammation, and structural changes in the lung parenchyma and airways. In addition to adult smokers, infants born prematurely represent a distinct high-risk population who can develop COPD later in life^[45]^. Indeed, significant epidemiological evidence, such as maternal smoking, the effects of viral infections, nutrition, and pollution, supports classifying COPD as a pediatric disease^[45]^. The pathogenesis of COPD involves a complex interplay of oxidative stress, protease-antiprotease imbalance, and immune dysregulation^[46]^. Concurrently, elevated protease activity, especially from matrix metalloproteinases (MMPs), leads to degradation of the extracellular matrix (ECM), compromising the structural integrity of the lungs^[47]^. Immune dysregulation further intensifies the disease process by promoting infiltration of inflammatory cells, thereby sustaining and worsening airway inflammation^[48]^. Together, these interconnected mechanisms drive the hallmark features of COPD: emphysema, chronic bronchitis, and remodeling of the small airways. Dysbiosis in the gut microbiota has been observed in patients with COPD and is associated with increased systemic inflammation^[49]^. Dysbiosis, as described above, can lead to increased gut permeability (“leaky gut”), allowing microbial products to enter systemic circulation and trigger systemic and pulmonary inflammation^[50]^. Preterm infants are at high risk of gut microbial dysbiosis due to immature immune systems and antibiotic use^[51]^. Microbial products such as short-chain fatty acids (SCFAs) produced in the gut can influence pulmonary immune responses, and conversely, lung inflammation can alter gut permeability and microbiota composition^[52]^.

### EVs as biomarkers in BPD and COPD

The potential of circulating EVs as diagnostic biomarkers in BPD and COPD is particularly promising^[53-56]^. EVs found in peripheral blood, bronchoalveolar lavage fluid (BALF), and even exhaled breath condensate reflect the molecular signature of diseased lung tissue, enabling early and less invasive detection, stratification, and monitoring of therapeutic responses^[2]^. In BPD, EVs enriched in pro-inflammatory miRNAs (e.g., miR-21, miR-155) and cytokines that can circulate systemically are produced through the activation of epithelial and immune cells. Interestingly, cord blood EVs in infants who develop BPD exhibit altered miRNA expression profiles linked to inflammation^[57]^. Interestingly, comparing EVs from neonates with and without BPD may help elucidate their contribution to disease pathogenesis and identify potential diagnostic biomarkers. For instance, EVs isolated from human umbilical cord neonates who later developed BPD exhibited significantly reduced cell proliferation, impaired capillary tube formation, and a more pronounced inhibition of endothelial cell migration in cultured human umbilical vein endothelial cells, compared to EVs from the non-BPD group^[58]^. miRNA profiling revealed significant differential expression of miR-103a-3p, miR-17-5p, miR-185-5p, miR-200a-3p, miR-20b-5p, and miR-765 between BPD- and non-BPD-derived EVs. Moreover, researchers have demonstrated the presence of microbiome-derived EVs in the urine of children with asthma, a disease that can be a consequence of BPD. These EVs were consistently altered, aligning with previous studies demonstrating changes in the lung and gut microbiomes. Urine may reflect specific patterns of EVs in the microbiome of children with asthma. Moreover, in these pediatric patients, EVs isolated from BALF present a unique phospholipid composition^[59]^. An interesting study displayed that umbilical cord blood EVs of patients with BPD exacerbate lung injury and disrupt lung angiogenesis in a hyperoxic mouse model. This negative impact can extend into adulthood. The authors proposed that EVs from umbilical cord cells of BPD patients may play a significant role in the progression of BPD and related lung diseases, suggesting that these EVs, along with their downstream genes and pathways, could serve as valuable predictive biomarkers and potential therapeutic targets for ventilator-induced pulmonary injury associated with BPD^[58]^.

Ransom *et al*. highlighted that EVs were enriched with the epithelial marker cluster of differentiation 24 (CD24) in preterm infants born in the late canalicular stage, indicating a possible role of CD24+ EVs in lung development. Furthermore, this study observed an increased level of CD14+ EVs, an emerging biomarker of severe disease, suggesting that CD14 may be a predictive marker of BPD^[60]^. In COPD, elevated levels of EVs have been detected in biological fluids such as sputum, BALF, and plasma. For instance, serological levels of endothelial-derived EVs (CD31+ or CD62E+) are increased in COPD patients and correlate with disease severity and vascular dysfunction^[61]^. Furthermore, Nieri *et al*. demonstrated that circulating EVs isolated from COPD patients exerted prothrombotic and pro-inflammatory activity *in vitro*^[62]^. Additionally, EV-associated miRNAs such as miR-21, miR-146a, and miR-223 have been implicated in regulating inflammatory pathways central to COPD pathogenesis. They may serve as accessible, non-invasive biomarkers for disease progression and response to therapy^[63]^. Notably, a recent study found elevated expression of EV-associated miR-21 in *in vitro* cultured COPD cells, suggesting its potential role in promoting cellular ageing in neighbouring cells^[64]^. Furthermore, miR-21 levels in EVs were found to be significantly upregulated in patients with COPD^[65]^.

### EVs as therapeutic tool in BPD and COPD

In recent years, the potential role of EVs in treating lung diseases has been rapidly growing^[66-68]^. In the context of pediatric diseases, the BPD preclinical model analyses the therapeutic efficiency of EVs^[69-71]^. The Kourembanas team assessed the efficacy of BM MSC-derived EVs as a treatment for a BPD animal model generated by exposing newborn mice to hyperoxia. Umbilical cord MSC-derived EVs demonstrated improvements in lung function, a decrease in fibrosis and pulmonary vascular remodelling, and a reduction in pulmonary hypertension. They also showed that the MSC-EVs’ mechanism of action is associated with the modulation of lung macrophage phenotype. Specifically, the macrophage M1-like inflammatory state was suppressed, and an increase in the M2-anti-inflammatory state was observed both *in vitro* and *in vivo* on alveolar macrophages from the neonatal murine hypoxia model^[72]^. On a different model of BPD, Porzionato *et al*. demonstrated that IT administration of clinical-grade MSC-EVs improved alveolarization parameters, pulmonary vascular remodelling, and inflammation in a rat model of BPD. They compared results between MSCs and MSC-EVs, showing that both treatments aimed to reduce hyperoxia-induced damage, with better results achieved using the EVs^[73]^. The studies mentioned above underlined that EV administration leads to better outcomes in alveolarization and lung vascularization compared to the use of mesenchymal stromal cells. This suggests that intratracheally administered EVs could be an effective approach to treat BPD, improving the morphometric parameters of alveolarization, pulmonary vascular remodelling, and inflammation, paving the way for clinical use in humans^[74]^. An elegant study in preterm lambs subjected to mechanical ventilation demonstrated that IV MSC-sEVs improved respiratory indices (oxygenation and ventilation), alveolar formation (radial alveolar count), capillary density, and vascular endothelial growth factor (VEGF)-R2 expression^[75]^. In newborn rats exposed to hyperoxia, both BM- and WJ-MSC EVs, delivered either intratracheally or intravenously, preserved alveolar and vascular structures, attenuated pulmonary hypertension, and exerted durable cardiopulmonary protective effects lasting up to 3 months post-treatment. Transcriptomic analysis revealed the upregulation of angiogenesis-associated gene sets, in addition to VEGF and hepatocyte growth factor (HGF)^[76]^. Mechanistic studies point to enhanced angiogenesis, modulation of macrophage phenotype (favoring anti-inflammatory M2), reduced oxidative stress, and preserved thymic and systemic immune architecture via cargo such as TNF-stimulated gene 6 protein (TSG 6), VEGF, and miRNAs, e.g., miR-425 activating Phosphoinositide 3-kinase/Protein kinase B (PI3K/AKT) by targeting Phosphatase and Tensin homolog (PTEN)^[77]^.

Along with BPD, EVs could offer a new therapeutic option for COPD patients, although so far, most efforts have focused on adults and not on preventing COPD in infants. For this reason, we selected the more relevant studies for the present review. For instance, in preclinical models of lung injury, MSC-EVs have shown immunomodulatory effects by reducing neutrophilic inflammation, suppressing cytokine production (TNF-α), and promoting epithelial repair^[78]^. Their cargo, particularly anti-inflammatory miRNAs (e.g., miR-126 and miR-30b), may suppress NF-κB signaling and modulate macrophage polarization toward a reparative phenotype^[79]^. In a chronic-induced murine model of COPD, administration of MSC-EVs led to significant improvements in lung function, including increased oxygen saturation, arterial pH, PaO_2_, and elevated IL-10 levels. Concurrently, treatment reduced the expression of pro-inflammatory cytokines (TNF-α, IL-12), decreased the total number of infiltrating pulmonary macrophages, and suppressed the antigen-presenting capacity of alveolar macrophages, as well as the activity of IL-17A-producing Natural Killer/Natural Killer T (NK/NKT) cells and neutrophils, thereby mitigating the inflammatory response.

Furthermore, MSC-exosomes were shown to downregulate the activation of both CD4^+^ and CD8^+^ T lymphocytes. Notably, inhalation of MSC-exosomes was associated with an improvement in exercise capacity, as reflected by increased walking distance, and an overall enhancement in quality-of-life indicators in the COPD model^[80]^. It is essential to underline that the use of EVs developed for BPD patients suggests that COPD development can be at least partially prevented. Notably, EVs from umbilical cord-derived or BM-derived MSCs have been shown to reduce alveolar simplification, preserve capillary density, and suppress inflammatory cytokine expression in neonatal rodent models exposed to hyperoxia. These findings suggest that EV-based therapies may replicate the beneficial effects of MSCs while minimizing the risks associated with cell-based treatments, such as immunogenicity and tumorigenicity. Early-phase clinical studies are underway to evaluate the safety and efficacy of EVs in neonatal lung disease, although challenges in standardization and large-scale production remain. EVs have shown therapeutic promise in preclinical models of COPD by modulating key pathogenic mechanisms, including oxidative stress, inflammation, and ECM degradation. EVs derived from MSCs have demonstrated the capacity to restore alveolar architecture, reduce neutrophilic inflammation, and suppress matrix MMP activity for tissue remodeling. Moreover, specific cargo molecules, such as anti-inflammatory miRNAs (e.g., miR-126, miR-146a) and growth factors (e.g., VEGF, HGF), are believed to contribute to these protective effects by reprogramming immune cells and enhancing epithelial regeneration.

Additionally, EVs may serve as precision delivery vehicles for therapeutic agents in COPD, offering targeted modulation of diseased tissues with minimal systemic toxicity. The engineering of EVs to carry anti-fibrotic or anti-inflammatory drugs is an area of active investigation and holds potential for personalized medicine approaches in COPD management. It is worth underlining that some important studies on adults could be applied to pediatric patients. Engineered MSC EVs are modified to enhance targeted delivery, traverse physiological barriers, and more effectively reach diseased lung tissue, thereby amplifying their therapeutic potential^[81]^. Additionally, engineered EVs demonstrate superior immunomodulatory functions; they can inhibit pro-inflammatory M1 macrophage polarization via modulation of miRNA content (e.g., reduced miR-21-3p levels), resulting in decreased secretion of TNF α, IL-6, and IL-12, key mediators in COPD pathogenesis^[82]^. These advances in EV engineering, including surface functionalization, gene or cargo loading, and parental-cell modifications, offer a versatile platform for the development of next-generation, targeted treatments for COPD and related chronic lung diseases.

### Clinical translation of stem cell-derived EVs in pediatrics - from bench to bedside

In recent years, EVs derived from stem cells have emerged as a promising therapeutic strategy for pediatric inflammatory and respiratory conditions. Several clinical trials are currently underway to evaluate their efficacy and safety. Table 2 provides a summary of selected trials, highlighting their key characteristics and current status. The information was sourced from the National Institutes of Health (NIH) Clinical Trials Database.

### Conclusion: translational relevance of extracellular vesicle applications and future perspectives

The intestine-lung axis constitutes a critical bidirectional communication network between the gastrointestinal and respiratory systems, with significant implications for host immunity, mucosal homeostasis, and disease pathogenesis (graphical abstract). The role of EVs is paramount under two different aspects: on the one hand, taking advantage of their anti-inflammatory properties, they can help gut microbiota-driven activation of innate and adaptive immune cells, such as T helper 17 cells, T regulatory cells, and dendritic cells, migrating systemically to play protective or regulatory roles in the lung^[83]^ [Table 1 and Figure 1]. On the other hand, EVs isolated in fluids, such as blood and saliva, help us understand the gut-lung axis also in early life, when microbial colonization and immune development co-evolve, shaping lifelong susceptibility to respiratory diseases. As a therapeutic tool, EVs may be delivered via inhalation (for lung inflammation), oral capsules (for gut dysbiosis), or intravenously, depending on the target compartment^[84]^. The preclinical data consistently show that EVs enhance intestinal stem cell survival, reducing pro-inflammatory signaling (e.g., NF-κB), and preserving epithelial barrier integrity. Innovations in EV engineering (e.g., cargo loading, surface modifications) may enhance specificity and efficacy for gut-lung axis restoration. Emerging evidence implicates gut microbiota-derived EVs as significant mediators of the gut-lung axis, potentially influencing the pathogenesis and clinical trajectory of both BPD and COPD^[85]^. Engineered EVs are rising as a promising therapeutic strategy due to their ability to be modified for targeted delivery to diseased tissues, while minimizing side effects on healthy cells. These modifications do not affect the size or morphology of EVs, enhance their accumulation at the target site, allow the loading of specific therapeutic molecules and improve their stability in the bloodstream, thereby extending their half-life. However, further research is needed to understand the pharmacodynamics, the *in vivo* metabolism and the interactions with the immune system (the mononuclear phagocyte system), before developing new therapeutics. To address the challenges of low yield and high production costs, alternative EV sources, such as plants, milk, and bacteria, are being explored. These naturally derived EVs have demonstrated therapeutic effects in preclinical models, including the control of inflammation, tissue regeneration, and immune modulation, offering cost-effective and scalable options for future clinical applications^[86,87]^.

**Figure 2:**
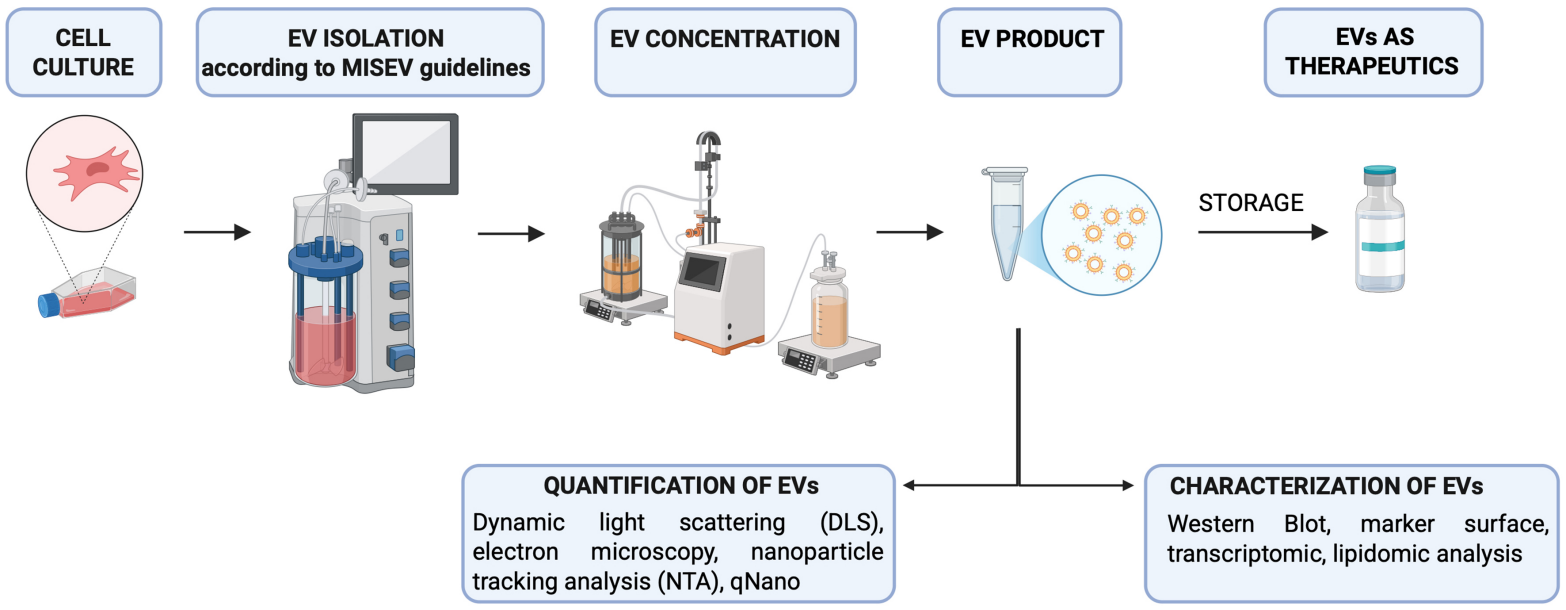
Schematic representation of the main steps for standardization of EV production as therapeutic tool. MISEV guidelines are highlighted in reference^[87]^. [Created in BioRender. Bisaccia, P. (2025) https://BioRender.com/37dge0q]. EVs: Extracellular vesicles; MISEV: Minimal Information for Studies of Extracellular Vesicles; DLS: dynamic light scattering; NTA: nanoparticle tracking analysis.

Note. These are the criteria that we followed for writing this review: We selected from PubMed all the articles that demonstrate a connection between the use of vesicles for pulmonary and intestinal diseases, with a particular focus on the pediatric field (keywords: extracellular vesicles in COPD, IBD, ARDS and pediatrics, extracellular vesicles in IBD and pediatrics, in NEC). Since studies in the pediatric field are limited, we included studies currently available on adults with the possibility of being translated into pediatrics.

In conclusion, EV-based therapies hold great promise within the field of advanced therapy medicinal products, as recognized by the European Medicines Agency (EMA). As these therapies progress toward clinical translation, particular emphasis is being placed on addressing key challenges such as production scalability, standardization of isolation and characterization [Figure 2]. These efforts are particularly critical for ensuring the safety, quality, and efficacy of exosome-based treatments in neonates and pediatric patients, people who present unique physiological vulnerabilities and therapeutic needs. Adherence to Good Manufacturing Practices (GMP) and ongoing technological advancements will be crucial in supporting the safe and effective use of these products in pediatric clinical settings.

## DECLARATIONS

### Copyright

© The Author(s) 2025.
